# Gene Analysis, Cloning, and Heterologous Expression of Protease from a Micromycete *Aspergillus ochraceus* Capable of Activating Protein C of Blood Plasma

**DOI:** 10.3390/microorganisms9091936

**Published:** 2021-09-11

**Authors:** Sergei K. Komarevtsev, Peter V. Evseev, Mikhail M. Shneider, Elizaveta A. Popova, Alexey E. Tupikin, Vasiliy N. Stepanenko, Marsel R. Kabilov, Sergei V. Shabunin, Alexander A. Osmolovskiy, Konstantin A. Miroshnikov

**Affiliations:** 1 Biology Department, Lomonosov Moscow State University, 119234 Moscow, Russia; veliania@gmail.com (E.A.P.); aosmol@mail.ru (A.A.O.); 2 All-Russian Scientific Research Veterinary Institute of Pathology, Pharmacology and Therapy, 394087 Voronezh, Russia; svshabunin@rambler.ru (S.V.S.); kmi@bk.ru (K.A.M.); 3 Shemyakin-Ovchinnikov Institute of Bioorganic Chemistry, Russian Academy of Sciences, 117997 Moscow, Russia; petevseev@gmail.com (P.V.E.); mm_shn@mail.ru (M.M.S.); svn@mx.ibch.ru (V.N.S.); 4 Institute of Chemical Biology and Fundamental Medicine, Siberian Branch of the Russian Academy of Sciences, 630090 Novosibirsk, Russia; niboch@niboch.nsc.ru (A.E.T.); kabilov@niboch.nsc.ru (M.R.K.)

**Keywords:** fibrinolytic protease, activator of protein C, micromycete, gene analysis, recombinant synthesis

## Abstract

Micromycetes are known to secrete numerous enzymes of biotechnological and medical potential. Fibrinolytic protease-activator of protein C (PAPC) of blood plasma from micromycete *Aspergillus ochraceus* VKM-F4104D was obtained in recombinant form utilising the bacterial expression system. This enzyme, which belongs to the proteinase-K-like proteases, is similar to the proteases encoded in the genomes of *Aspergillus fumigatus* ATCC MYA-4609, *A. oryzae* ATCC 42149 and *A. flavus* 28. Mature PAPC-4104 is 282 amino acids long, preceded by the 101-amino acid propeptide necessary for proper folding and maturation. The recombinant protease was identical to the native enzyme from micromycete in terms of its biological properties, including an ability to hydrolyse substrates of activated protein C (pGlu-Pro-Arg-pNA) and factor Xa (Z-D-Arg-Gly-Arg-pNA) in conjugant reactions with human blood plasma. Therefore, recombinant PAPC-4104 can potentially be used in medicine, veterinary science, diagnostics, and other applications.

## 1. Introduction

Fibrinolytic proteases that can dissolve thrombi have attracted considerable attention because of their potential therapeutic application. These enzymes have been found in various sources, including bacteria, fungi, snake venoms, earthworms, marine creatures, and others. Since such proteases have performed well as candidates for the treatment of cardiovascular diseases, currently, new enzymes are being actively sought to satisfy the needs of the pharmaceutical industry [1,2,3].

Soil micromycete *Aspergillus ochraceus* VKM-F4104D produces an extracellular fibrinolytic protease capable of activating protein C of blood plasma [4,5]. This important anticoagulant factor, which inhibits the formation of thrombin, stimulates fibrinolysis, and acts as a cytoprotective signal molecule, is produced in the liver as non-active zymogen [6]. The activation of protein C is required for the diagnostics and therapy of cardiovascular diseases [7]. Physiologically, it is a complex thrombin-catalysed process, which occurs on the surface of endothelial cells and involves two membrane receptors, thrombomodulin and endothelial protein C receptor [8]. Activators from snake venoms, especially from the *Agkistrodon contortrix contortrix* snake, which directly converts protein C into active form, are widely used in diagnostics and medicine, especially, in chromogenic and clotting protein C assays [9,10]. PAPC from *A. ochraceus* VKM-F4104D (PAPC-4104) has been shown to possess a similar ability to activate protein C, so potentially, it can be applied in practice as an easily available, less expensive alternative to activators from snake venoms [11,12]. However, despite the affordability of micromycete cultivation, the yield of the secreted target protein is low [4,5], and the purification of native PAPC is a complicated multi-step procedure. The production of recombinant PAPC-4104 may be a valuable solution promoting the application of this enzyme. To complete this task, it is necessary to overcome the fragmentary knowledge on transcription, folding, and secretion of this protein in the host micromycete. In this paper, we report the analysis of *A. ochraceus* VKM-F4104D gene encoding PAPC-4104 and the phylogenetic and evolutionary characterisation of the enzyme, followed by cloning and expression of the functional PAPC-4104 in an *E. coli* system.

## 2. Materials and Methods

### 2.1. Microorganism and Growth Conditions

*Aspergillus ochraceus* VKM-F4104D was isolated from a soil sample collected from the Krasnodar region, Russia, and routinely grown on wort–agar slants (4% wort, 1.8% agar) at 28 °C. Spores from seven-day old micromycete were washed from the culture surface to obtain seed material. Spore suspension was inoculated into the growth medium (6.7% wort, 1% glucose, 0.1% peptone, pH 5.5–6.0) and cultivated for two days. Subsequently, part of the biomass was transferred into the fermentation medium (3.5% glucose, 1% fish meal hydrolysate, 0.2% NaCl, 0.125% starch, 0.1% peptone, 0.05% KH_2_PO_4_, 0.05% MgSO_4_, pH 5.5–6.0) and grown for three days. Cultivation was performed using 750 mL Erlenmeyer flasks containing 100 mL of a nutrient medium on a rotary shaker at 200 rpm at 28 °C.

### 2.2. cDNA Sequencing and Cloning of PAPC-4104

Mycelial biomass after a first stage of cultivation (100 mg) was frozen in liquid nitrogen, and total RNA was isolated using a PureLink RNA Mini Kit (Thermo Fisher Scientific, Waltham, MA, USA) and an On-Column DNase I Digestion Set (Merck, Darmstadt, Germany) according to the manufacturer’s instructions. To obtain mRNA of *A. ochraceus*, a poly(A)+ RNA was isolated from total RNA using a NebNext Poly(A) mRNA Magnetic Isolation Module, following library preparation with NEBNext Ultra Directional RNA Library Prep Kit for Illumina (New England Biolabs, Ipswich, MA, USA). Library sequencing was performed on a Miseq genome sequencer (2 × 300 cycles, Illumina) in the SB RAS Genomics Core Facility (ICBFM SB RAS, Novosibirsk, Russia). After the trimming of adapters and quality filtering of reads by Trim Galore v0.4.2 (https://github.com/FelixKrueger/TrimGalore accessed on 30 May 2021), the transcriptome was assembled using rnaSPAdes v3.9.0 (Center for Algorithmic Biotechnology, St.-Petersburg, Russia) [13]. Using a BLAST search with the MEROPS database [14], a contig of 1686 nt in length was identified.

Total cDNA of putative PAPC-4104 was amplified from poly(A)+ RNA using the Verso 1-Step RT-PCR Hot-Start Kit (Thermo Fisher Scientific, Waltham, MA, USA) with primers flanking the protein coding sequence Asp-21F (5′-TCTCATCATCACACAGCTTCTG-3′) and Asp-1348R (5′-TGTCGTCCCAAGTAACTCTAGC-3′). The same primers were used to amplify the PAPC-4104 gene from genomic DNA to reveal the possible splicing sites. PCR products were separated by electrophoresis in 1.0% agarose gel, and the target band was cut and isolated from the gel slice using a Monarch DNA Gel Extraction Kit (New England Biolabs, Ipswich, MA, USA). Sanger sequencing of PCR products was performed on an ABI 3130xl Genetic Analyser with a BigDye Terminator Cycle Sequencing Kit v3.1 (Thermo Fisher Scientific, Waltham, MA, USA). Amplified cDNA PCR product was used in subsequent subcloning to the expression vector.

### 2.3. In Silico Protein Analysis

The amino acid sequence of PAPC-4104 was obtained from cDNA using EMBOSS Translation Tools (https://www.ebi.ac.uk/Tools/st/emboss_transeq/ accessed on 30 May 2021) and analysed with NCBI BLAST (https://blast.ncbi.nlm.nih.gov/Blast.cgi accessed on 15 May 2021). A protein domain search was conducted with an InterPro server (http://www.ebi.ac.uk/interpro) employing PHOBIUS (http://phobius.sbc.su.se accessed on 20 May 2021), Pfam (https://pfam.xfam.org accessed on 20 May 2021), and PRINTS protein fingerprints databases. Secondary structure prediction was carried out by garnier (http://www.bioinformatics.nl/cgi-bin/emboss/garnier accessed on 20 May 2021). The presence of a signal peptide was determined with the SignalP 5.0 server (www.cbs.dtu.dk/services/SignalP accessed on 25 May 2021) and PHOBIUS transmembrane topology and signal peptide predictor (http://phobius.sbc.su.se accessed on 20 May 2021). Protein remote homology detection was performed on the HHpred server (https://toolkit.tuebingen.mpg.de accessed on 25 May 2021) [15]. The tertiary structure prediction was made with AlphaFold 2.0 [16] with default settings and visualised with PyMOL v.2 [17]. The model quality assessment was performed with ProQ3D server (https://proq3.bioinfo.se/pred/ accessed on 20 May 2021) [18] and ModFOLD8 server (https://www.reading.ac.uk/bioinf/ModFOLD/ModFOLD8_form.html accessed on 25 May 2021) [19]. The predicted structure was superimposed with the HHpred highest-score fungal protease from *Lecanicillium psalliotae* sequence (PDB ID: 3F7M) using the functionality of PyMOL.

### 2.4. Alignments and Phylogeny

Gene sequences were downloaded from the NCBI GenBank (ftp://ftp.ncbi.nlm.nih.gov/genbank accessed on 15 May 2021) to the Geneious 2020 (Biomatters Ltd., Auckland, New Zealand) working environment (http://www.geneious.com accessed on 16 May 2021) [20]. The search for homologs was conducted by BLAST (https://blast.ncbi.nlm.nih.gov/Blast.cgi accessed on 15 May 2021) using NCBI online and custom-made databases. Coding sequences found were translated and searched against protein databases to ensure the presence of a subtilisin-like (peptidase S8) domain, using an InterPro server (https://www.ebi.ac.uk/interpro/ accessed on 20 May 2021). Amino acid sequences were aligned with Clustal Omega [21] with auto settings and trimmed using trimAL (http://trimal.cgenomics.org/trimal accessed on 25 May 2021) with the “automated1” parameter optimised for maximum likelihood phylogenetic tree reconstruction. Phylogenetic trees were constructed using the maximum likelihood (ML) method with an RAxML program [22] with a GAMMA I BLOSUM62 protein model, and the robustness of the trees was assessed by bootstrapping (1000). The alignments and trees were visualised with assistance of Geneious 2020.

### 2.5. Construction of the Expression Plasmid

The cDNA fragment encoding the propeptide and mature protein of PAPC-4104 (pro-PAPC: Ala^21^ to Ala^404^) was PCR-amplified using primers 5′-TAAGAAGGAGATATACCATGGCTCCCGTCGAGAACACC and 5′-TGGTGGTGGTGCTCGAGAGCAGCGCCGTTGTAGGCA. The amplified DNA fragment was digested with NcoI and XhoI and ligated into the corresponding restriction sites of pET23d (+) vector (Novagen Calbiochem, Madison, WI, USA) in order to construct the plasmid for expression of the pro-PAPC-4104 with C-terminal hexahistidine tag.

### 2.6. Expression and Purification of Recombinant PAPC-4104

A clone of *E. coli* BL21 (DE3) with pET23d-pro-PAPC-4104 plasmid was incubated at 37 °C in a Lysogeny Broth (LB) medium, supplemented with 100 μg/mL until OD600–0.6. Then, isopropyl-β-D-thiogalactopyranoside (IPTG) was added to a final concentration of 0.5 mM, with further incubation for 16 h at 16 °C. The cells were harvested by centrifugation at 4000× *g* at 4 °C for 15 min, resuspended in 20 mM Tris-HCl buffer pH 8.0, 200 mM NaCl, and 1 mM CaCl_2_ and disrupted by sonication (Branson, Brookfield, CT, USA). The crude lysate was centrifuged at 10,000× *g* at 4 °C for 15 min, filtered through the 0.45 μm filter (Merck, Darmstadt, Germany), and applied to the 10 mL (1.6 × 6 cm) column with Ni-NTA Superflow (GE Healthcare, Chicago, IL, USA). Proteins were eluted from the column by stepwise gradient of imidazole (0-20-60-200 mM) in 20 mM Tris-HCl, 200 mM NaCl, 1 mM CaCl_2_, pH 8.0 buffer. Protein content in the flow was monitored by absorbance at 280 nm.

### 2.7. Purification of Native PAPC-4104

To compare the properties of recombinant and native PAPC-4104, the enzyme was isolated and purified from *A. ochraceus* VKM-F4104D fermentation medium, as described previously [23]. Briefly, the 500 mL of medium after cultivation of micromycete was filtered through the filter paper for the purpose of removing biomass, and ammonium sulfate was added to the filtrate to 70% saturation. The precipitate was separated by centrifugation at 15,000× *g* at 4 °C for 20 min, dissolved in 50 mM Tris-HCl buffer pH 8.0, 1 mM CaCl_2_, containing ammonium sulfate at 35% saturation, re-centrifuged to remove insoluble debris, and loaded into a 5 mL (1.6 × 3 cm) phenyl-sepharose column (GE Healthcare, Chicago, IL, USA). PAPC-4104 was eluted with a 35 to 0% ammonium sulfate saturation descending gradient, pumped through a 5 mL (1.6 × 3 cm) DEAE-sepharose column (Pharmacia, Uppsala, Sweden) previously equilibrated with 50 mM Tris-HCl buffer pH 8.0, 1 mM CaCl_2_, concentrated using a Centricon-10 centrifugal concentrator (Merck, Darmstadt, Germany), and further purified by gel filtration on a Sephadex G50 (Pharmacia, Uppsala, Sweden). Protein content in the flow was monitored by absorbance at 280 nm.

### 2.8. Enzyme Activity Assays

The activity was qualitatively assayed by the droplet method using chromogenic peptide substrates for activated protein C (pGlu-Pro-Arg-pNA), factor Xa (Z-D-Arg-Gly-Arg-pNA), plasmin (H-D-Val-Leu-Lys-pNA), thrombin (Tos-Gly-Pro-Arg-pNA, H-D-Phe-Pip-Arg-pNA), and kallikrein (H-D-Pro-Phe-Arg-pNA) (Sigma-Aldrich, Burlington, MA, USA) after preliminary incubation with and without human blood plasma, as described previously [4,11]. For the analysis, 20 μL of PAPC-4104 sample was mixed with 5 μL of human plasma diluted twice or 50 mM Tris-HCl buffer, 1 mM CaCl_2_, pH 8.0. The resulting mixture was incubated at room temperature. After that, 10 μL of 0.05% solution of a chromogenic substrate in 50 mM Tris-HCl buffer, 1 mM CaCl_2_, pH 8.0 was added and incubated for 5 min under the same conditions. The results of reactions were manifested by a yellow colouration and confirmed by measuring of optical density at 405 nm using NanoDrop One spectrophotometer (Thermo Fisher Scientific, Waltham, MA, USA). If measured values exceeded 0.1, the substrate specificity of enzyme estimated as positive (“+”); if not, it was negative (“−”).

Kinetic parameters were determined by addition of 100 μL of chromogenic peptide substrate Tos-Gly-Pro-Arg-pNA in increasing concentrations (0.065, 0.125, 0.25, 0.5, 1, 2 mg/mL) to a reaction cuvette containing 100 μL of enzyme (2 μg/mL) and 150 μL of 50 mM Tris-HCl buffer pH 8.0, 1 mM CaCl_2_. The experiments were performed at 25 °C, and absorbance at 405 nm was monitored using a BioSpectrometer kinetic spectrophotometer (Eppendorf, Hamburg, Germany). The Michaelis constant (Km), catalytic constant (kcat), and the maximum rate of the reaction (Vmax) catalysed by enzymes were calculated through a double reciprocal (Lineweaver–Burk) plot using Microsoft Excel.

### 2.9. General Analytical Methods

The protein concentration of obtained fractions was determined by measuring optical density at 280 nm using NanoDrop One spectrophotometer (Thermo Fisher Scientific, Waltham, MA, USA). The analysis of protein components of fractions was performed using 12.5% SDS-PAGE with Coomassie Blue R250 staining.

## 3. Results

### 3.1. Gene Organization and Protein Sequence

Extracellular alkaline proteases secreted by micromycetes of the *Aspergillus* genus show a high degree of homology to subtilisin-like serine proteases [24,25]. In the MEROPS database, subtilases are classified as members of the S8A subfamily within the serine peptidases family S8 and belong to the SB clan [26]. Extracellular subtilisins are initially expressed with an N-terminal signal sequence with an adjacent auto-inhibitory domain, named the inhibitor I9 domain or prodomain, required for correct folding of the mature enzyme, and peptidase S8 domain [27,28].

**Figure 1:**
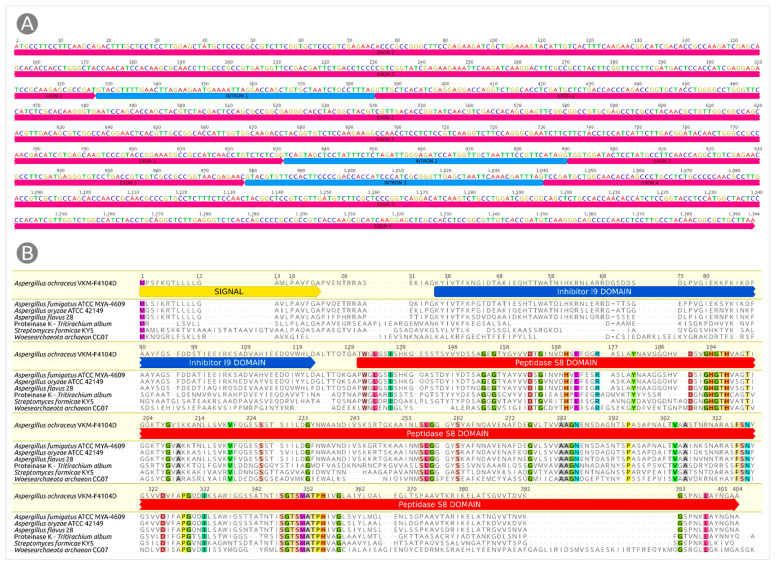
(**A**) The gene of *Aspergillus ochraceus* VKM-F4104D alkaline protease. Exons are coloured crimson and introns are coloured blue. (**B**) The protein sequence of *Aspergillus ochraceus* VKM-F4104D alkaline protease (upper row) aligned with subtilase sequences from different organisms of various species. Conservative amino acid residues are highlighted with colours. Signal peptide region (yellow strip), inhibitor I9 domain (blue strip) and peptidase S8 domain (red strip) are annotated on the basis of bioinformatic analysis and experimentally found corresponding regions in *Aspergillus fumigatus* ATCC MYA-4609, *Aspergillus oryzae* ATCC 42,149, and *Aspergillus flavus* 28 homologs.

The gene sequence for PAPC-4104 (NCBI GenBank accession # MW183406) is shown in Figure 1A. The gene involves three non-coding regions and four translated exons. The complete translated 404 amino acid-long sequence for PAPC-4104 is shown in Figure 1B. Bioinformatic analysis demonstrated the presence of a 21 amino acid secretory signal sequence Met^1^-Ala^21^ (likelihood 0.9917) and a cleavage site between position 21 and 22 (likelihood 0.9287). Signal peptide provides the secretion of a native protein across the endoplasmic reticulum lumen and is further removed by a signal peptidase during translocation. The hydrophobic core of signal peptide was predicted to comprise nine amino acid residues. Even though signal peptides are extremely heterogeneous [29], a BLAST search showed the presence of homologous sequences among other *Aspergillus* genomes and protease coding sequences. The database search confirmed the presence of I9 inhibitor and S8 peptidase domains (Figure 1B). The I9 domain (the middle part of the precursor, Pro^22^-Asp^122^) also acts as an intramolecular chaperon essential for the correct folding of the polypeptide chain. It is autocleaved and degraded during maturation. Protease domain Ala^123^-Ala^404^ provides the catalytic activity of the mature enzyme. Such a domain structure is common for subtilisin-like proteases.

### 3.2. Tertiary Structure and Active Site Residue

**Figure 2:**
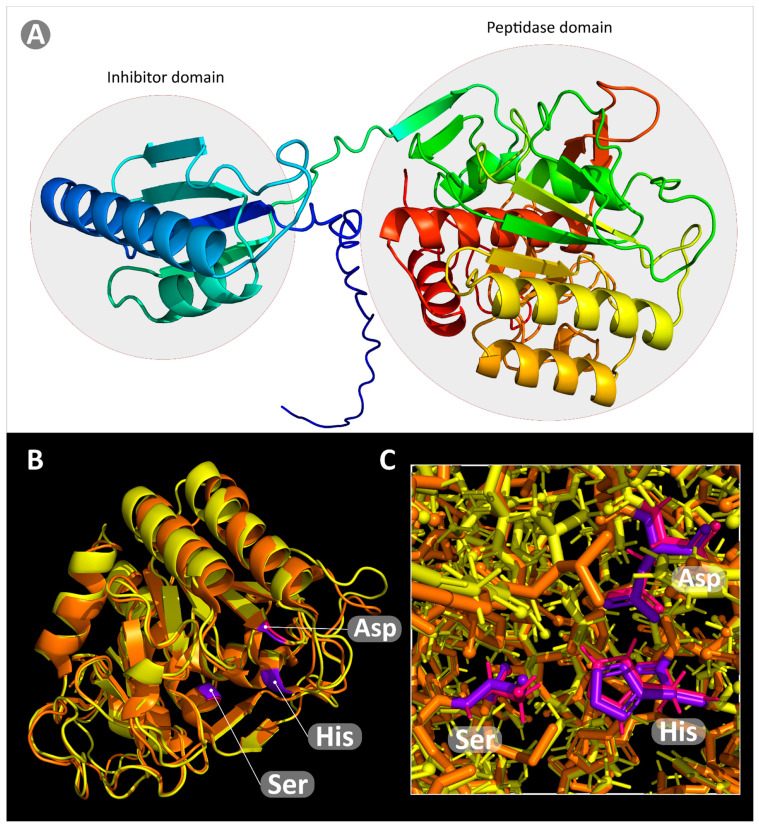
(**A**) Predicted structure of alkaline protease from *Aspergillus ochraceus* VKM-F4104D. The model is coloured based on a rainbow gradient scheme, where the N-terminus of the polypeptide chain is coloured blue and the C-terminus is coloured red. The structure was predicted with AlphaFold 2.0 [16], and the model with the AlphaFold highest confidence was taken. (**B**,**C**) Superimposition of the peptidase domains of the predicted structure of *Aspergillus ochraceus* VKM-F4104D protease (yellow) and the experimentally found structure of *Lecanicillium psalliotae* protease (PDB ID: 3F7M) (orange) using PyMOL (score 705, RMSD 0.502) [17]. The catalytic residues are coloured hot pink (*Aspergillus ochraceus*) and purple–blue (*Lecanicillium psalliotae*).

Subtilisin-like serine proteases are single-domain α/β-proteins. They have different substrate-binding clefts and only have similarly positioned key amino acid residues of the catalytic sites. These proteases perform their catalytic role using three key residues—Ser, His, and Asp—which are commonly referred to as the catalytic triad [30]. Some subtilases may also contain a conserved catalytic residue of asparagine (Asn) in the catalytic domain [30,31]. Protein remote homology detection performed on the HHpred server pointed to *Lecanicillium psalliotae* protease [32] and to similar highest-score fungal proteases. The tertiary structure of PAPC-4104 was predicted with AlphaFold 2.0 (Figure 2A). The model quality assessment with ProQ3D demonstrated the global model quality ProQ3D score of 0.715. The model quality assessment with ModFOLD8 demonstrated the global model quality score of 0.603 and a high quality of the prediction of the peptidase domain structure with a predicted residue error of 1–3 Å (Supplementary Figure S1A,B).

The predicted structure of the peptidase domain was superimposed with the experimentally found structure of *Lecanicillium psalliotae sequence* protease and revealed the catalytic triad to be composed of Ser-350, His-194, and Asp-41 amino acid residues (Figure 2B,C).

### 3.3. Phylogeny and Evolution

**Figure 3:**
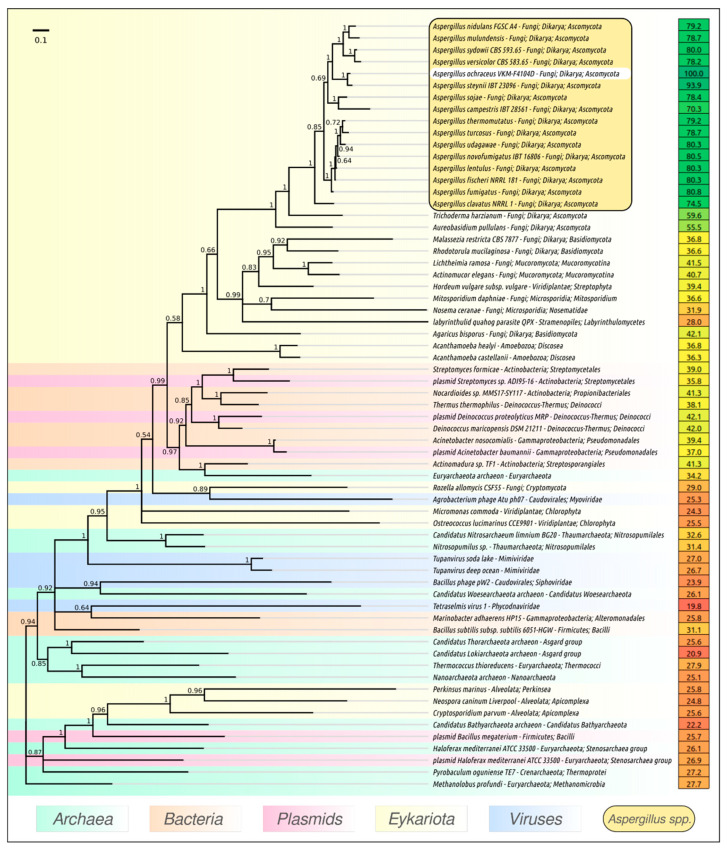
Phylogenetic tree obtained with MrBayes based on alkaline protease protein sequences found with a BLAST search of NCBI databases. Bayesian posterior probabilities are indicated above their branch. Taxonomic classification is taken from NCBI sequence attributes and is shown to the right of the organism name. The percentage of amino acid identity compared to *Aspergillus ochraceus* VKM-F4104D alkaline protease is shown in the right column. The scale bar shows 0.1 estimated substitutions per site and the tree was rooted to *Methanolobus profundi*; of 2,000,000 generations, every 200 generations were sampled, with an average standard deviation of split frequencies of 0.0091.

Subtilisin-like proteases are found in all kingdoms of cellular life, as well as in many viral genomes [33]. A BLAST search using NCBI databases indicated the presence of subtilisins in bacterial mobilome. Subtilisin-like proteases are ubiquitous in fungal genomes, suggesting a diverse fungal lifestyle; subtilisin-like peptidase domain (S8) was found in all 21 families of fungi [34]. The phylogenetic tree (Figure 3) demonstrates the closeness of proteases identified in the genomes of *Aspergillus* sp. The subtilisin closest evolutionally appears to be the protease of *Aspergillus steynii* IBT 23096 (pairwise identity 93.9%, Figure 3). Among the classic representatives of the subtilase family, PAPC-4104 has noticeable homology (41%) with proteinase K from *Tritirachium album* (UniProt P06873) [35] (Figure 1B). A closer similarity to PAPC-4104 was observed for elastinolytic alkaline protease from pathogenic *Aspergillus fumigatus* ATCC MYA-4609, (P28296, 80%) [36], alkaline protease from industrial *Aspergillus oryzae* ATCC 42149 (P12547, 77%) [37,38], and elastinolytic alkaline protease from pathogenic *Aspergillus flavus* 28 (P35211, 74%) [39] (Figure 1B).

The topology of the fungal part of the subtilisin phylogenetic tree (Figure 3) generally resembles the topology of the fungi phylogenetic trees obtained by the analysis of conservative genes [40,41]. The presence of subtilisin genes in bacterial genomes and plasmids and their phylogenetic placement close to homologous genes from *Cryptomycota* and *Amoboezoa* can testify to the important role of horizontal gene transfer in the early evolution of serine proteases. The widespread presence of genes for serine proteases in plasmids can be explained by their significant role as possible factors of virulence. It has been shown that subtilisin-like proteases contribute to the high virulence of different pathogens [42,43,44,45]. Interestingly, phylogenetic analysis can also indicate the relatedness of subtilisins belonging to different viral groups. The evolutionary study of viral serine proteases is beyond the scope of the present research but can be conducted in future work.

### 3.4. Properties of the Recombinant PAPC-4104

**Figure 4:**
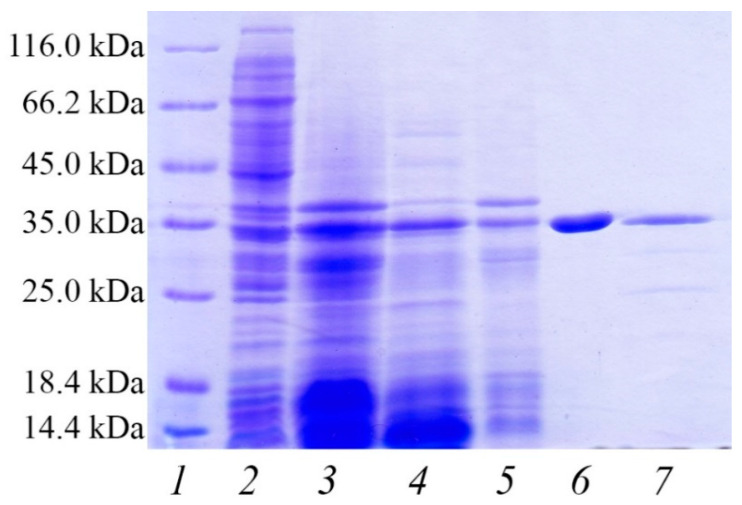
SDS-PAGE analysis of expression, folding and purification of alkaline protease from *A. ochraceus* VKM-F4104D (PAPC): 1—protein molecular weight marker, 2—uninduced *E. coli* BL21 (DE3), 3—induced *E. coli* BL21 (DE3), 4—supernatant of centrifugation after sonication (soluble fraction), 5—pellet of centrifugation after sonication (insoluble fraction), 6—soluble fraction of PAPC after purification with Ni-NTA affinity chromatography, 7—native PAPC after purification.

Since signal peptide is capable of obstructing recombinant gene expression in *E. coli* [46], the corresponding fragment of PAPC-4104 was removed in the design of the expression construct. The gene for recombinant expression under the control of the T7 promoter contains the propeptide and protease domain (from Ala^21^ to Ala^404^) of PAPC-4104 with C-terminal six histidine residues. The calculated molecular mass of His-tagged non-mature pro-PAPC-4104 is 41.3 kDa, and it is 29.6 kDa for mature PAPC-4104. The size of the native enzyme estimated from the electrophoretic mobility is 33 kDa [11]. Overnight expression at 16 °C for 16 h yielded an accumulation of protein with the size of 33–34 kDa, predominantly in soluble form (Figure 4, lanes 2–5). This means that the synthesis and maturation of the target protein was successful at 16 °C. The activity of the resulting protein was indirectly supported by the proteolytic degradation of domestic *E. coli* proteins (Figure 4, lane 3). Subsequent purification with Ni-NTA affinity column chromatography made it possible to obtain PAPC-4104 in homogeneous form from cell lysate (Figure 4, lane 6). The use of SDS-PAGE to estimate the size of PAPC-4104 compared to its calculated molecular weight was in agreement with data obtained for homologous enzymes in other studies [36,37,39]. Both recombinant and native PAPC demonstrated the same electrophoretic mobility (Figure 4, lanes 6, 7).

The recombinant enzyme had properties similar to those of a native protein: it was able to hydrolyse substrates of activated protein C (pGlu-Pro-Arg-pNA) and factor Xa (Z-D-Arg-Gly-Arg-pNA) in conjugant reactions with human blood plasma (Table 1). Additionally, it was capable of cleaving chromogenic peptide substrates of plasmin (H-d-Val-Leu-Lys-pNA) and thrombin (Tos-Gly-Arg-pNA) in direct reactions, and there was no cleavage with other studied substrates. This is similar to proteases from snake venoms, with the ability to activate target proenzymes, protein C, and factor X; alongside the simplicity of obtaining the recombinant form of PAPC-4104, this makes it a good alternative to snake venom activators [9,10,11].

To ensure the enzymatic quality of the recombinant enzyme we have performed the estimation of basic kinetic parameters of native and recombinant PAPC-4104 using a model chromogenic substrate Tos-Gly-Pro-Arg-pNA in ambient conditions. The assay yielded equal Vmax of 34.9 nmol/min for native and 34.4 nmol/min for recombinant PAPC-4104, and close values for kcat (57 and 56 s^−1^, correspondingly), while the values for Km were 285 μM for native and 435 μM for recombinant enzyme (Supplementary Figure S2). The Km value is approximately 10-fold higher than one normally shown for thrombin cleavage of this substrate. However, considering Tos-Gly-Pro-Arg-pNA as a valid but non-specific target for PAPC-4104, we can conclude that the kinetic properties of native and recombinant enzymes are fairly close.

## 4. Discussion

Many micromycetes are well known as producers of proteases targeting proteins of the haemostatic system. So, in addition to the fibrinolytic properties of such proteases, the ability of some to activate a number of blood coagulation factors, such as plasminogen [47,48], protein C [11], prothrombin [49], prekallikrein [50], and factor X [51], is known. Proteolytic enzymes of these micromycetes are capable of cleaving a characteristic range of chromogenic peptide substrates of proteins of the haemostasis system and demonstrate differences in the activity with respect to globular and fibrillar proteins. They have different optima of pH and temperature and differ in isoelectric point and molecular weight. Many of listed proteases are produced by f representatives of the genus *Aspergillus*. Such enzymes can find practical application both in the composition of thrombolytic drugs for therapy and as components of a diagnostic kit for determining the content of these proteins in the blood. Protease-activators of protein C and factor X, produced by *Aspergillus ochraceus*, have shown their effectiveness for the determination of the content of these proenzymes in vitro in comparison with commercial analog-protease-activators from snake venom [12,51]. It is assumed that these proteases can become a more affordable alternative for the development of diagnostic kits for the detection of blood clotting diseases in humans and animals.

Extracellular proteases of filamentous fungi with the indicated types of activity are subtilisin-like alkaline proteases that could benefit not only diagnostic and medical, but many veterinary and biotechnological applications. In particular, extracellular protease of *A. ochraceus* (PAPC-4104) possesses substrate specificity similar to snake venom protein C activators. However, the production of this protein through fungal cultivation results low yields [4,5] and the purification of PAPC-4104 is difficult due to excessive pigment contamination [23]. Recombinant production of similar fungal alkaline proteases is also hindered by the multidomain nature and complicated translation, folding, and maturation of the target protein [35,36,37,38,39]. The phylogenetic and evolutionary relationship of the enzyme with other subtilisins was of particular interest.

In this research, PAPC of micromycete *A. ochraceus* VKM-F4104D was obtained in recombinant form, utilising the bacterial expression system. This enzyme, which belongs to the proteinase-K-like proteases, is similar (more than 74%) to the proteases encoded in the genomes of *A. fumigatus* ATCC MYA-4609, *A. oryzae* ATCC 42149 and *A. flavus* 28. We reported the analysis of *A. ochraceus* VKM-F4104D gene encoding PAPC-4104 and showed that mature PAPC-4104 is 282 amino acids long, preceded by the 101-amino acid propeptide necessary for proper folding and maturation.

Additionally, bioinformatic analysis of the gene and mRNA sequences enabled us to design a strategy to obtain PAPC-4104 in a functional and soluble form in the simple *E. coli* expression system. Due to primary biochemical property studies, the recombinant protease was identical to the native enzyme from *A. ochraceus* VKM-F4104D in terms of its biological properties, including an ability to hydrolyse chromogenic peptide substrates of activated protein C (pGlu-Pro-Arg-pNA) and factor Xa (Z-D-Arg-Gly-Arg-pNA) in conjugant reactions with human blood plasma. The native and recombinant PAPC had similar molecular weight and demonstrated similar electrophoretic mobility.

Thus, obtained recombinant PAPC-4104 can potentially be used in medicine, veterinary science, diagnostics, and other applications.

## Figures and Tables

**Table 1:** Substrate specificity of recombinant and native PAPC with chromogenic peptide substrates. If measured values of A_405_ of a quantitative reaction exceeded 0.1, the substrate specificity of enzyme is estimated as positive (“+”); if not, as negative (“−”).

Substrate	Activity of Recombinant PAPC-4104	Activity of Native PAPC-4104
**Conjugate reactions (with blood plasma)**		
pGlu-Pro-Arg-pNA	+	+
Z-D-Arg-Gly-Arg-pNA	+	+
**Direct reactions (without blood plasma)**		
pGlu-Pro-Arg-pNA	−	−
Z-D-Arg-Gly-Arg-pNA	−	−
H-D-Val-Leu-Lys-pNA	+	+
Tos-Gly-Pro-Arg-pNA	+	+
H-D-Pro-Phe-Arg-pNA	−	−
H-D-Phe-Pip-Arg-pNA	−	−

## Data Availability

The gene sequence for PAPC-4104 is submitted to the NCBI GenBank database and is available under the accession number MW183406.

## Supplementary Materials

The following are available online at https://www.mdpi.com/article/10.3390/microorganisms9091936/s1. Figure S1: (**A**) ModFOLD8 residue error plot. (**B**) ModFOLD8 3D view of per-residue accuracy of the *Aspergillus ochraceus* protease model. The model is coloured based on a rainbow gradient scheme, where the residues with the lowest predicted residue errors are coloured blue and the residues with the highest predicted residue errors are coloured red. The N-terminus of the polypeptide chain is labeled “N” and the C-terminus is labeled “C”. Figure S2: Lineweaver-Burk plot for native (**A**) and recombinant (**B**) form of PAPC-4104. [Tos]—concentration of chromogenic peptide substrate Tos-Gly-Pro-Arg-pNA. The error bars represent standard deviation.

## References

[1] Altaf F., Wu S., Kasim V. (2021). Role of fibrinolytic enzymes in anti-thrombosis therapy. Front. Mol. Biosci..

[2] Kumar S.S., Sabu A. (2019). Fibrinolytic enzymes for thrombolytic therapy. Adv. Exp. Med. Biol..

[3] Kotb E. (2014). The biotechnological potential of fibrinolytic enzymes in the dissolution of endogenous blood thrombi. Biotechnol. Prog..

[4] Osmolovskiy A.A., Kreier V.G., Kurakov A.V., Baranova N.A., Egorov N.S. (2012). *Aspergillus ochraceus* micromycetes-producers of extracellular proteinases-protein C activators of blood plasma. Appl. Biochem. Microbiol..

[5] Osmolovsky A.A., Kreier V.G., Baranova N.A., Kurakov A.V., Egorov N.S. (2013). Production of extracellular proteinases-protein C activators of blood plasma-by the micromycete *Aspergillus ochraceus* during submerged and solid-state fermentation. Appl. Biochem. Microbiol..

[6] Bouwens E.A.M., Stavenuiter F., Mosnier L.O. (2013). Mechanisms of anticoagulant and cytoprotective actions of the protein C pathway. J. Thromb. Haemost..

[7] Mohammed S., Favaloro E.J. (2017). Laboratory testing for activated protein C resistance (APCR). Methods Mol. Biol..

[8] Chen K., Stafford A.R., Wu C., Yeh C.H., Kim P.Y., Fredenburgh J.C., Weitz J.I. (2017). Exosite 2-directed ligands attenuate protein C activation by the thrombin-thrombomodulin complex. Biochemistry.

[9] Asmat A., Ramzan F. (2018). Venom protein C activators as diagnostic agents for defects of protein C system. Protein Pept. Lett..

[10] Gempeler-Messina P.M., Volz K., Bühler B., Müller C. (2001). Protein C activators from snake venoms and their diagnostic use. Haemostasis.

[11] Osmolovskiy A.A., Kreyer V.G., Baranova N.A., Kurakov A.V., Egorov N.S. (2015). Properties of extracellular proteinase—an activator of protein C in blood plasma formed by *Aspergillus ochraceus*. Appl. Biochem. Microbiol..

[12] Osmolovskiy A.A., Orekhova A.V., Kreyer V.G., Baranova N.A., Egorov N.S. (2018). Possibility of application of extracellular protease of micromycete *Aspergillus ochraceus* VKM F-4104D for determination of protein C content in human blood plasma. Biomed. Khim.

[13] Bushmanova E., Antipov D., Lapidus A., Prjibelski A.D. (2019). RnaSPAdes: A de novo transcriptome assembler and its application to RNA-Seq data. GigaScience.

[14] Rawlings N.D., Barrett A.J., Thomas P.D., Huang X., Bateman A., Finn R.D. (2018). The MEROPS database of proteolytic enzymes, their substrates and inhibitors in 2017 and a comparison with peptidases in the PANTHER database. Nucleic Acids Res..

[15] Zimmermann L., Stephens A., Nam S.Z., Rau D., Kübler J., Lozajic M., Gabler F., Söding J., Lupas A.N., Alva V. (2018). A Completely Reimplemented MPI Bioinformatics Toolkit with a New HHpred Server at its Core. J. Mol. Biol..

[16] Jumper J., Evans R., Pritzel A., Green T., Figurnov M., Ronneberger O., Tunyasuvunakool K., Bates R., Žídek A., Potapenko A. (2021). Highly accurate protein structure prediction with AlphaFold. Nature.

[17] The PyMOL Molecular Graphics System, Version 2.0 Schrödinger, LLC.

[18] Uziela K., Hurtado D., Shu N., Wallner B., Elofsson A. (2017). ProQ3D: Improved model quality assessments using Deep Learning. Bioinformatics.

[19] McGuffin L., Aldowsari F., Alharbi S., Adiyaman R. (2021). ModFOLD8: Accurate global and local quality estimates for 3D protein models. Nucleic Acids Res..

[20] Kearse M., Moir R., Wilson A., Stones-Havas S., Cheung M., Sturrock S., Buxton S., Cooper A., Markowitz S., Duran C. (2012). Geneious Basic: An integrated and extendable desktop software platform for the organization and analysis of sequence data. Bioinformatics.

[21] Sievers F., Wilm A., Dineen D., Gibson T.J., Karplus K., Li W., Lopez R., McWilliam H., Remmert M., Söding J. (2011). Fast, scalable generation of high quality protein multiple sequence alignments using Clustal Omega. Mol. Syst. Biol..

[22] Stamatakis A. (2014). RAxML version 8: A tool for phylogenetic analysis and post-analysis of large phylogenies. Bioinformatics.

[23] Komarevtsev S.K., Popova E.A., Kreyer V.G., Miroshnikov K.A., Osmolovskiy A.A. (2020). Purification of the protease activator of protein C of human blood plasma produced by the micromycete *Aspergillus ochraceus* VKM F-4104D. Appl. Biochem. Microbiol..

[24] Morya V.K., Yadav S., Kim E.K., Yadav D. (2012). In silico characterization of alkaline proteases from different species of *Aspergillus*. Appl. Biochem. Biotechnol..

[25] Morya V.K., Yadav V.K., Yadav S., Yadav D. (2016). Active site characterization of proteases sequences from different species of *Aspergillus*. Cell Biochem. Biophys..

[26] Rawlings N.D., Barrett A.J. (1994). Families of serine peptidases. Methods Enzymol..

[27] Bryan P.N. (2002). Prodomains and protein folding catalysis. Chem. Rev..

[28] Eder J., Fersht A.R. (1995). Pro-sequence-assisted protein folding. Mol. Microbiol..

[29] Von Heijne G. (1985). Signal sequences. The limits of variation. J. Mol. Biol..

[30] Dodson G., Wlodawer A. (1998). Catalytic triads and their relatives. Trends Biochem. Sci..

[31] Siezen R.J., Leunissen J.A. (1997). Subtilases: The superfamily of subtilisin-like serine proteases. Protein Sci..

[32] Liang L., Meng Z., Ye F., Yang J., Liu S., Sun Y., Guo Y., Mi Q., Huang X., Zou C. (2010). The crystal structures of two cuticle–degrading proteases from nematophagous fungi and their contribution to infection against nematodes. FASEB J..

[33] Siezen R.J., De Vos W.M., Leunissen J.A., Dijkstra B.W. (1991). Homology modelling and protein engineering strategy of subtilases, the family of subtilisin-like serine proteinases. Protein Eng. Des. Sel..

[34] Muszewska A., Stepniewska-Dziubinska M.M., Steczkiewicz K., Pawlowska J., Dziedzic A., Ginalski K. (2017). Fungal lifestyle reflected in serine protease repertoire. Sci. Rep..

[35] Gunkel F.A., Gassen H.G. (1989). Proteinase K from *Tritirachium album Limber* Characterization of the chromosomal gene and expression of the cDNA in *Escherichia coli*. Eur. J. Biochem..

[36] Jaton-Ogay K., Suter M., Crameri R., Falchetto R., Fatih A., Monod M. (1992). Nucleotide sequence of a genomic and a cDNA clone encoding an extracellular alkaline protease of *Aspergillus fumigatus*. FEMS Microbiol. Lett..

[37] Tatsumi H., Ogawa Y., Murakami S., Ishida Y., Murakami K., Masaki A., Kawabe H., Arimura H., Nakano E., Motai H. (1989). A full length cDNA clone for the alkaline protease from *Aspergillus oryzae*: Structural analysis and expression in *Saccharomyces cerevisiae*. MGG Mol. Gen. Genet..

[38] Cheevadhanarak S., Renno D.V., Saunders G., Holt G. (1991). Cloning and selective overexpression of an alkaline protease-encoding gene from *Aspergillus oryzae*. Gene.

[39] Ramesh M.V., Sirakova T., Kolattukudy P.E. (1994). Isolation, characterization, and cloning of cDNA and the gene for an elastinolytic serine proteinase from *Aspergillus flavus*. Infect. Immun..

[40] Wang H., Xu Z., Gao L., Hao B. (2009). A fungal phylogeny based on 82 complete genomes using the composition vector method. BMC Evol. Biol..

[41] James T.Y., Kauff F., Schoch C.L., Matheny P.B., Hofstetter V., Cox C.J., Celio G., Gueidan C., Fraker E., Miadlikowska J. (2006). Reconstructing the early evolution of Fungi using a six-gene phylogeny. Nature.

[42] Bonifait L., De La Cruz Dominguez-Punaro M., Vaillancourt K., Bart C., Slater J., Frenette M., Gottschalk M., Grenier D. (2010). The cell envelope subtilisin-like proteinase is a virulence determinant for *Streptococcus suis*. BMC Microbiol..

[43] Yin S., Li M., Rao X., Yao X., Zhong Q., Wang M., Wang J., Peng Y., Tang J., Hu F. (2016). Subtilisin-like protease-1 secreted through type IV secretion system contributes to high virulence of *Streptococcus suis 2*. Sci. Rep..

[44] Gao B.J., Mou Y.N., Tong S.M., Ying S.H., Feng M.G. (2020). Subtilisin-like Pr1 proteases marking the evolution of pathogenicity in a wide-spectrum insect-pathogenic fungus. Virulence.

[45] Ksiazek M., Karim A.Y., Bryzek D., Enghild J.J., Thøgersen I.B., Koziel J., Potempa J. (2015). Mirolase, a novel subtilisin-like serine protease from the periodontopathogen *Tannerella forsythia*. Biol. Chem..

[46] Ni H., Guo P.C., Jiang W.L., Fan X.M., Luo X.Y., Li H.H. (2016). Expression of nattokinase in *Escherichia coli* and renaturation of its inclusion body. J. Biotechnol..

[47] Sharkova T.S., Matveeva E.O., Kreier V.G., Osmolovskiy A.A., Kurakov A.V., Baranova N.A., Egorov N.S. (2016). Production of proteinase-plasminogen activators by micromycete *Tolypocladium inflatum* k1. Appl. Biochem. Microbiol..

[48] Kornienko E.I., Osmolovskiy A.A., Kreyer V.G., Baranova N.A., Kotova I.B., Egorov N.S. (2021). Characteristics and properties of the complex of proteolytic enzymes of the thrombolytic action of the micromycete *Sarocladium strictum*. Appl. Biochem. Microbiol..

[49] Liu C., Matsushita Y., Shimizu K., Makimura K., Hasumi K. (2007). Activation of prothrombin by two subtilisin-like serine proteases from *Acremonium* sp.. Biophys. Res. Commun..

[50] Zvonareva E.S., Osmolovskiy A.A., Kreyer V.G., Baranova N.A., Kotova I.B., Egorov N.S. (2015). Identification of targets for extracellular proteases activating proteins of the haemostatic system produced by micromycetes *Aspergillus ochraceus* and *Aspergillus terreus*. Russ. J. Bioorg. Chem..

[51] Orekhova A.V., Osmolovskiy A.A., Kreyer V.G., Baranova N.A., Egorov N.S. (2019). Possibility for application of extracellular protease of micromycete *Aspergillus ochraceus* for determining factor X content in human blood plasma. Mosc. Univ. Biol. Sci. Bull..

