# Supplementary material for: Gene Analysis, Cloning, and Heterologous Expression of Protease from a Micromycete Aspergillus ochraceus Capable of Activating Protein C of Blood Plasma
Source: Microorganisms. 2021 Sep 11;9(9):1936. doi: 10.3390/microorganisms9091936 (PMC8471544; doi:10.3390/microorganisms9091936)
Supplement: Supplementary file 1 [file microorganisms-09-01936-s001.zip › microorganisms-1335413-Supplementary.pdf]

microorganisms

Supplementary Figure S1

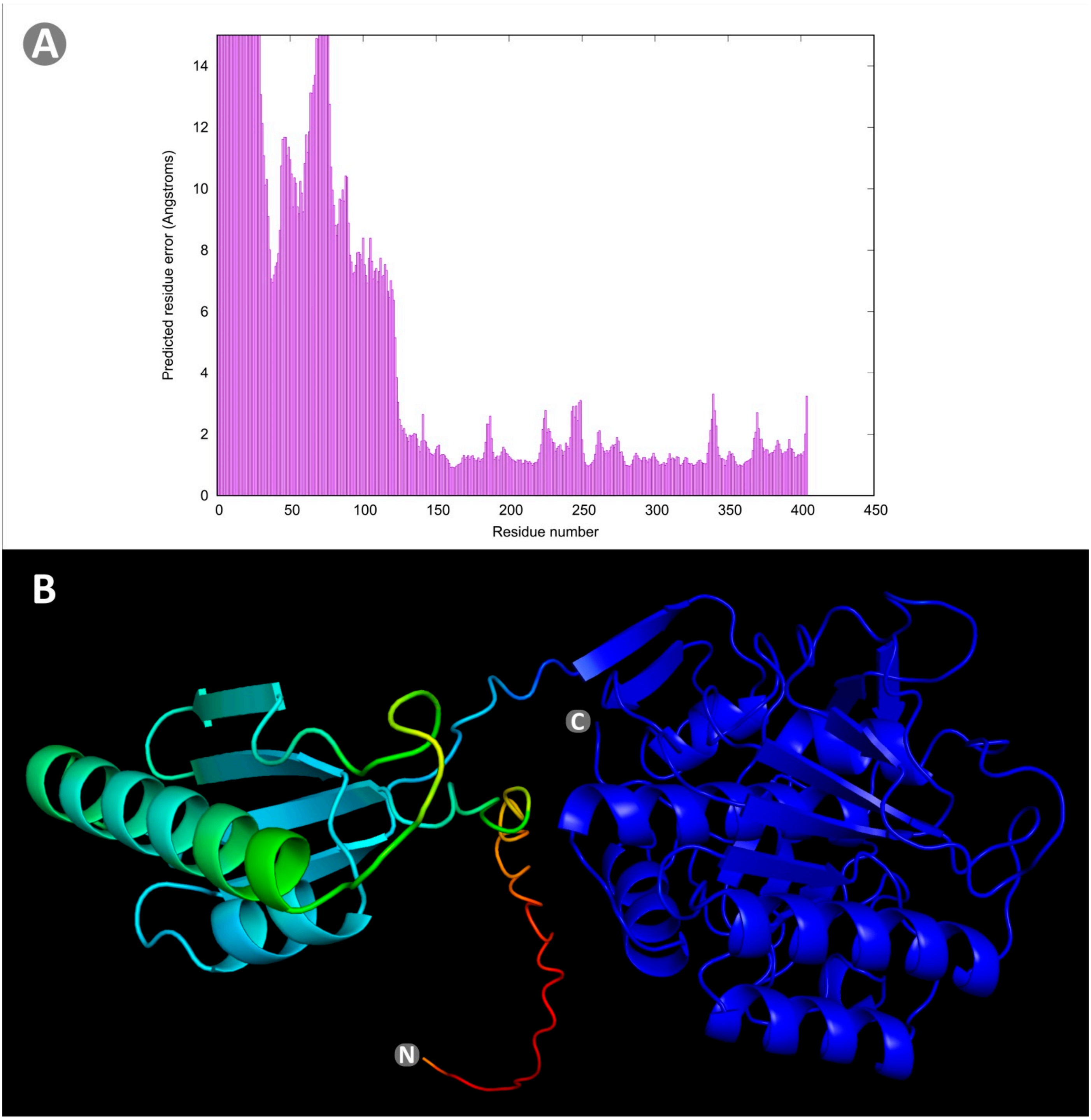

Supplementary Figure S1. (**A**) ModFOLD8 residue error plot. (**B**) ModFOLD8 3D view of per-residue accuracy of the *Aspergillus ochraceus* protease model. The model is coloured based on a rainbow gradient scheme, where the residues with the lowest predicted residue errors are coloured blue and the residues with the highest predicted residue errors are coloured red. The N-terminus of the polypeptide chain is labeled "N" and the C-terminus is labeled "C".

Supplementary Figure S2

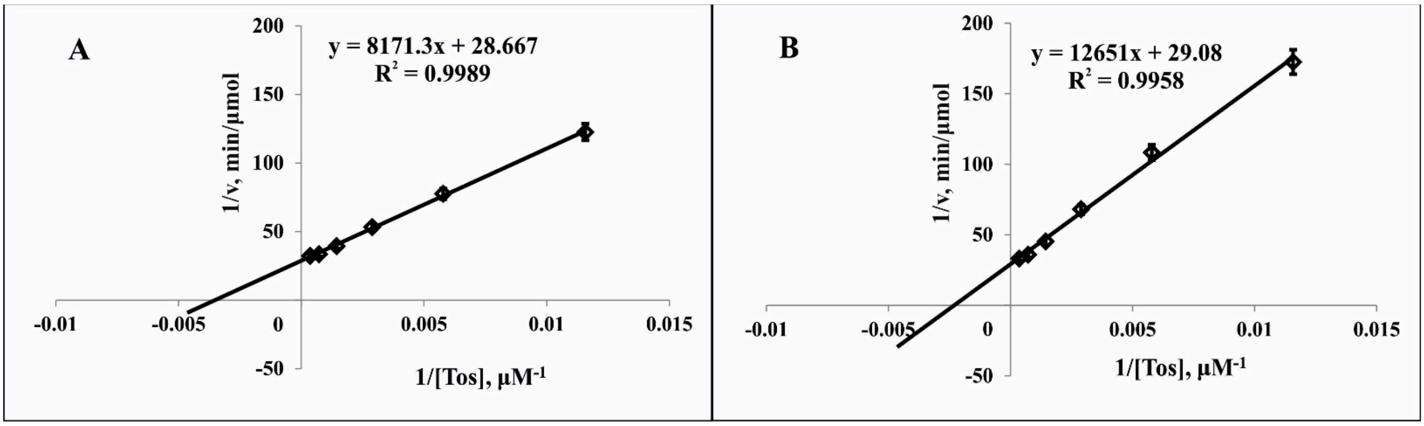

Supplementary Figure S2. Lineweaver-Burk plot for native (**A**) and recombinant (**B**) form of PAPC-4104. [Tos] – concentration of chromogenic peptide substrate Tos-Gly-Pro-Arg-pNA The error bars represent standard deviation.
